# Health status, quality of life, and satisfaction of patients awaiting multidisciplinary bariatric care

**DOI:** 10.1186/1472-6963-12-139

**Published:** 2012-06-08

**Authors:** Raj S Padwal, Sumit R Majumdar, Scott Klarenbach, Daniel W Birch, Shahzeer Karmali, Linda McCargar, Konrad Fassbender, Arya M Sharma

**Affiliations:** 1 Department of Medicine, University of Alberta, 2F1.26 Walter C. Mackenzie Health Sciences Centre, 8440-112th Street, Edmonton, AB T6G 2B7, Canada; 2 Department of Surgery and CAMIS (Center for the Advancement of Minimally Invasive Surgery), Room 502, Community Services Centre, 10240 Kingsway Ave NW, Royal Alexandra Hospital, Edmonton, AB T5H 3V9, Canada; 3 Department of Agricultural, Food and Nutritional Sciences, 2-021D Li Ka Shing Centre for Health Research Innovation, University of Alberta, Edmonton, AB T6G 2E1, Canada; 4 Department of Oncology, University of Alberta, Room 329, Evironmental Engineering Bldg, NW Corner of 112th Street and 87 Ave, Edmonton, Alberta T6G 2G2, Canada

**Keywords:** Canada, Bariatric care, Wait list, Quality-of-life, Health services research

## Abstract

**Background:**

Protracted, multi-year wait times exist for bariatric care in Canada. Our objective was to examine wait-listed patients’ health status and perceptions regarding the consequences of prolonged wait times using a cross-sectional study design nested within a prospective cohort.

**Methods:**

150 consecutive consenting subjects wait-listed for multi-disciplinary bariatric assessment in a population-based medical/surgical bariatric program were surveyed. Health status was measured using a visual analogue scale (VAS). A Waiting List Impact Questionnaire (WLIQ) examined employment, physical stress, social support, frustration, quality of life, and satisfaction with care. Multivariable linear regression analysis adjusted for age, sex and BMI identified independent predictors of lower VAS scores.

**Results:**

136 (91%) subjects were women, mean age was 43 years (SD 9), mean BMI was 49.4 (SD 8.3) kg/m^2^ and average time wait-listed was 64 days (SD 76). The mean VAS score was 53/100 (SD 22). According to the WLIQ, 47% of subjects agreed/strongly agreed that waiting affected their quality of life, 65% described wait times as ‘concerning’ and 81% as ‘frustrating’. 86% reported worsening of physical symptoms over time. Nevertheless, only 31% were dissatisfied/very dissatisfied with their overall medical care. Independent predictors of lower VAS scores were higher BMI (beta coefficient 0.42; p = 0.03), unemployment (13.7; p = 0.01) and depression (10.3; p = 0.003).

**Conclusions:**

Patients wait-listed for bariatric care self-reported very impaired health status and other adverse consequences, attributing these to protracted waits. These data may help benchmark the level of health impairment in this population, understand the physical and mental toll of waiting, and assist with wait list management.

**Trial registration:**

Clinicaltrials.gov NCT00850356

## Background

Morbid obesity (defined herein as a body mass index [BMI] of ≥ 35 kg/m^2^) affects 8.9% of Canadians and has tripled in prevalence from 1978-79 to 2007-09 [1,2]. Premature mortality, obesity-related comorbidity, diminished quality of life and higher health care costs are more likely to afflict morbidly obese individuals compared to their normal weight counterparts [3,4]. Managing the complications and encouraging treatment of obesity has become a major priority and a public health concern.

Current clinical practice guidelines emphasize the complex, chronic nature of obesity and stress the importance of lifelong, sustainable lifestyle change [5,6]. They also identify a multidisciplinary approach to the assessment and management of obese individuals as the most effective and the preferred treatment approach [5,6]. Medical therapy consists primarily of intensive lifestyle modification (diet, exercise and behavioural modification counselling) and is recommended for all obese individuals, including those that are morbidly obese [5,6]. Accordingly, broad multidisciplinary team expertise in medicine, nutrition, physical activity and mental health is required to optimally deliver lifestyle modification [6]. In addition, according to these guidelines, bariatric surgery should be considered in patients refractory to non-surgical therapy who have either severe obesity (BMI ≥40 kg/m^2^) or moderate obesity (BMI 35.0-39.9 kg/m^2^) and a major obesity related comorbidity (e.g., hypertension, sleep apnea, diabetes) [6,7].

Weight management interventions are infrequently delivered in the primary care setting, and providers cite several reasons for this including a lack of training to deliver weight management interventions, a lack of access to multidisciplinary allied health team support and a relative paucity of effective interventions [8]. Comprehensive multidisciplinary medical and surgical obesity bariatric care within Canada’s publicly funded health care system is thus usually delivered by multidisciplinary bariatric specialty clinics. However, access to such care is limited because of high demand, limited capacity, lengthy wait lists and protracted, multiyear wait times [9]. For example, estimated wait times for surgery in Canada average 5 years [10] and wait times for the medical and surgical bariatric care of morbidly obese individuals within a regional program in Alberta, Canada average 2-3 years [11]. In both Canada and England, well under 1% of potentially eligible individuals received bariatric surgery in 2009-10 [7,9,11]. UK bariatric surgeons have characterized access in their country as ‘inconsistent, unethical and completely dependent upon geographic location’ and some primary care trusts within the UK appear to be limiting surgeries to patients with BMI levels above 50 kg/m^2^ in an attempt to ration the number of procedures performed and reduce wait list volumes [7,12,13]. In stark contrast to the wait times experienced by bariatric patients, benchmark wait times for other elective surgeries in Canada such as orthopedic procedures, coronary bypass and cataract removal are ≤16 weeks [14]. In 80% of cases, Canadians are receiving these procedures within benchmark times [14].

Medical and surgical treatments for obesity can reduce weight and medical comorbidity and improve health-related quality of life (QOL); improvements in these outcomes are especially large following surgery [15-18]. Thus, lengthy wait times are widely considered by bariatric specialists to be detrimental to physical and mental health [9]. However to our knowledge no prior study has assessed patients’ perspectives on this matter, and data examining health status and patients’ views regarding the impact and consequences of waiting are nonexistent. The purpose of this study was to assess these patient-reported or humanistic outcomes in a representative sample of patients wait-listed for a bariatric (i.e., medical and surgical) care in a population based regional obesity program.

## Methods

### Subjects and setting

One hundred and fifty consecutive consenting adult (age ≥18 years) subjects wait-listed for assessment in the Edmonton Weight Wise Clinic were surveyed. Edmonton Weight Wise is a regional obesity program established 2005 to deliver integrated, patient-focused, evidence-based care to the Edmonton Zone of Alberta Health Services (AHS) [11]. The Edmonton Zone is one of the largest integrated health delivery regions in Canada, serving a catchment population of approximately 1.6 million residents within greater Edmonton. Weight Wise consists of a central, region-wide, single-point-of-access referral system; community education and weight management sessions; and adult and pediatric bariatric specialty clinics. The adult specialty clinic provides both medical and surgical treatment to practitioner-referred patients 18 years of age or greater with BMI levels of 35 kg/m^2^ or greater who have been unsuccessful with prior attempts at managing chronic obesity. Approximately 800 new referrals are seen and approximately 200 bariatric surgeries are performed annually. We estimate that over 125 000 adult patients within the Weight Wise catchment area have a BMI ≥ 35 kg/m^2^[11].

**Figure 1:**
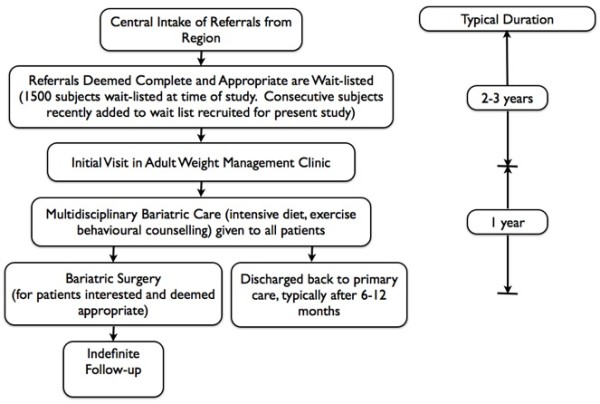
Structure of the Edmonton Weight Wise Program.

After new referrals to the adult clinic are confirmed to be complete and appropriate, patients then are designated as ‘wait-listed’ and wait in queue until they are granted an initial clinic visit in the adult specialty clinic. Subjects involved in the present study were recruited shortly after they were wait-listed (within months) and were waiting for their initial assessment in the adult specialty clinic (Figure 1). All patients entering the adult clinic undergo a multidisciplinary obesity evaluation and medical management program (e.g., intensive lifestyle modification, mental health assessment, screening for obstructive sleep apnea and eating disorders, physiotherapy and social worker assessment if needed), typically lasting 4-6 months. Patients interested in surgery also undergo a multidisciplinary assessment during this period to determine if they are appropriate for this procedure and, if approved for surgery, typically undergo the procedure after 3-6 months of additional wait. Patients not interested in surgery continue received intensive medical management for an additional six months. At the time this study was conducted, the time from referral processing to the initial clinic visit averaged over two years and over 1500 patients were wait listed.

### Study cohort

The 150 subjects included in the present analysis comprise the wait-list arm of the Alberta Population-based Prospective Evaluation of the Quality of Life Outcomes and Economic Impact of Bariatric Surgery (APPLES) study. Details with respect to this study, including technical appendices, have been previously published [11]. In summary, APPLES is a 500-patient, population-based, two-year prospective controlled study designed to assess the impact of extended wait-times for bariatric care and examine the clinical and cost-effectiveness of bariatric treatment in the Canadian context. In APPLES, 150 bariatric surgery subjects, 200 medical subjects and 150 wait-listed subjects were enrolled in consecutive fashion between January 2009-February 2010. At the time of interview and data collection, wait-listed subjects were facing wait times of approximately 2-3 years before their initial assessment in the adult clinic.

Outcomes are reported in the entire 150-patient wait-listed sample and in the subgroup of subjects indicating a ‘strong’ or ‘very strong’ interest in undergoing bariatric surgery (n = 96) to facilitate generalizability to programs in which patients are wait-listed for both surgical and medical bariatric care, as well as primarily surgical treatment.

The University of Alberta Research Ethics Board approved this study and informed consent was obtained from all subjects.

### Data collection and measurements

Subjects were asked to rate their overall state of health from 0-100 using a visual analogue scale (VAS) with 100 reflecting the ‘best imaginable state of health’ [19]. Satisfaction with medical care was assessed in a similar manner to other studies conducted in this health region [20,21] using two previously validated items taken from the Patient Satisfaction Questionnaire [22], which were scored on a 5-point Likert Scale and both positively and negatively worded to reduce acquiescent response bias:

a. The medical care I have been receiving is just about perfect

b. I am dissatisfied with some things about the medical care I received.

The Waiting List Impact Questionnaire (WLIQ) [23], adapted from a study of wait times for coronary revascularization was administered to assess the perceived impact of protracted wait times on health status. The WLIQ originally consisted of a series of 47 statements identified through an open-ended patient interview process, each scored using a 5-point Likert scale, that examined the impact of waiting on 5 domains (employment, physical stress, social support, frustration and quality of life). Seven cardiac surgery-specific items deemed not relevant to the bariatric setting were eliminated [23]. One statement that assessed the subject’s degree of interest in bariatric surgery was incorporated in with the remaining 39 items constituted the 40-item WLIQ used herein; the modified WLIQ questionnaire has been previously published in its entirety [11].

We collected basic sociodemographic information and clinical data at the time of program intake (i.e., soon after they were wait-listed). Subjects with a history of hypertension, blood pressure levels of greater than 140/90 mm Hg at the baseline visit [11], or subjects receiving antihypertensive drug treatment were considered hypertensive. Patients with a history of type 2 diabetes, on treatment with antidiabetic medications or who had a single fasting glucose measurement of 7.0 mmol/L or greater were classified with diabetes. Depression was diagnosed based upon a self-reported history or treatment with antidepressant medications. The presence of all other comorbidities was determined by self-report.

### Analysis

Descriptive analyses, consisting of means, medians, and proportions were conducted. For ease of presentation, responses to the WLIQ and patient satisfaction items were collapsed from five categories into three – strongly agree/agree, neutral and disagree/strongly disagree.

Multivariable linear regression was used to identify independent predictors of health status according to the VAS in all 150 subjects. Age, sex, and BMI (per unit increase) were first forced into all models. Additional covariates with a p-value < 0.20 on univariate analysis were also considered in the initial model. Potential model covariates included all the variables listed in Table 1. The final model was created using a stepwise backwards selection method to determine which of these additional covariates contributed to the model at a Wald Chi-square p-value of 0.2. SAS (Version 9.2, Cary, NC) and SPSS (PSW 18, Somers, NY) were used for all analyses.

**Table 1:** Baseline Demographics

**Variable**	**All patients (n = 150)**	**Subgroup Interested in Surgery (n = 96)**
	**Mean or No. (SD or %)**	**Mean or No. (SD or %)**
Mean age (years)	43 (9)	44 (9)
Female Sex	136 (91)	89 (93)
Weight (kg)	134.7 (25.1)	135.5 (24.7)
Body Mass Index (kg/m^2^)	49.4 (8.3)	49.9 (8.4)
Time on Wait List (days)	64 (76)	59 (74)
Smoking		
Never	62 (41)	34 (35)
Former	64 (43)	41 (43)
Current	24 (16)	21 (22)
Marital Status		
Married/Common Law	80 (53)	46 (48)
Divorced/Separated	22 (15)	18 (19)
Single	48 (32)	32 (33)
Education		
No high school	1 (1)	0 (0)
Some high school	16 (11)	8 (8)
High school diploma	26 (17)	16 (17)
Some post-secondary	27 (18)	13 (14)
Post-secondary graduate	80 (53)	59 (62)
Annual Income		
Less than $15 000	9 (6)	6 (6)
$15 000 – 29 999	17 (11)	9 (9)
$30 000 – 49 999	22 (15)	16 (17)
$50 000 – 79 999	45 (30)	27 (28)
$80 000 or greater	52 (35)	33 (34)
Not answered	5 (3)	5 (5)
Employment Status		
Full-time	90 (60)	59 (62)
Part-time	18 (12)	12 (13)
Casual/volunteer	1 (1)	1 (1)
Long-term disability	12 (8)	9 (9)
Homemaker	9 (6)	5 (5)
Unemployed	15 (10)	8 (8)
Retired	5 (3)	4 (4)
Other	5 (3)	3 (3)
Race		
Caucasian	138 (92)	90 (94)
Hispanic	1 (1)	1 (1)
First Nations	2 (1)	2 (2)
South Asian	2 (1)	2 (2)
Other	6 (4)	1 (1)
Type 2 Diabetes	35 (23)	22 (23)
Hypertension	70 (47)	45 (47)
Dyslipidemia	38 (25)	27 (28)
Coronary Artery Disease	6 (4)	6 (6)
Peripheral Vascular Disease	2 (1)	0 (0)
Cerebrovascular Disease	1 (1)	1 (1)
Congestive Heart Failure	0 (0)	0 (0)
Sleep Apnea	44 (29)	32 (33)
On CPAP	19 (13)	13 (14)
Gastroesophageal Reflux	57 (38)	44 (46)
Osteoarthritis	44 (29)	31 (32)
Polycystic Ovarian Syndrome	20 (13)	16 (17)
Hypothyroidism	32 (21)	25 (26)
Depression	93 (62)	68 (71)
Fibromyalgia	18 (12)	15 (16)
Anxiety	69 (46)	48 (50)

## Results

### Baseline characteristics

The pre-defined sample size of 150 was reached after 425 subjects had been telephoned (overall response rate 35%). The 275 subjects that did not participate had demographic characteristics similar to the respondents. 240 (89%) were female, with a mean age of 42.9 (SD 9.3) years and a mean BMI of 47.2 kg/m^2^ (SD 7.1).

Baseline sociodemographic characteristics of the participants are detailed in Table 1. The mean number of days on the wait list at the time of survey was 64 (SD 76).

### Overall health status

The average overall state of health score according to the VAS was 53/100 (SD 22) in the overall sample and 49/100 (SD 22) in the subgroup interested in bariatric surgery. In the final multivariable analyses, the independent correlates of lower VAS scores were higher body mass index (beta coefficient 0.42; p = 0.03), unemployment (13.7; p = 0.01), and depression (10.3; p = 0.003) (Table 2). Sleep apnea was of borderline significance (6.6; p = 0.09). Overall model R [2] was 0.20.

**Table 2:** Linear Regression Analysis Examining Predictors of the Visual Analogue Scale (n = 150)*

**Predictor Variable**	**Beta-coefficient**	**P value**	**Beta-coefficient**	**P value**
	**(Univariable)**		**(Multivariable)**	
Age (years)	-0.08	0.68	0.11	0.52
Female sex	13.4	0.03	8.34	0.16
Body mass index (kg/m^2^)	-0.57	0.008	-0.42	0.03
Time on Waitlist (days)	-0.01	0.32		
Smoker (former or current)	10.3	0.03		
Married or common law (vs. all other categories)	-2.9	0.42		
Post-secondary graduate (vs. all other education levels)	4.7	0.24		
Low income, ≤$30, 000 (vs. all other income categories)	-3.45	0.47		
Employed full or part-time (vs. all other categories)	17.7	<0.0001	13.7	0.0003
Type 2 diabetes	-3.6	0.34		
Hypertension	-5.3	0.17		
Dyslipidemia	-2.7	0.51		
Coronary artery disease	-6.4	0.48		
Sleep apnea	-12.9	0.0009	-6.55	0.095
Gastroesophageal reflux	-7.2	0.05		
Osteoarthritis	-9.4	0.02		
Polycystic ovarian syndrome	1.78	0.74		
Hypothyroidism	-0.37	0.93		
Depression	-12.18	0.0009	-10.28	0.003
Fibromyalgia	-8.73	0.12		
Anxiety	-6.54	0.07		

*Predictor variables with p values < 0.2 were candidates for final model. Age, BMI and sex are forced in final model, which was constructed using a backward selection procedure.

### Waiting list impact questionnaire

Responses to all 40 items are summarized in Table 3 and responses according to each domain are outlined below.

**Table 3:** Waiting List Impact Questionnaire Results

**Statement**	**No. of Patients With Response (%)**					
	**All patients (n = 150)**			**Subgroup Interested in Surgery (n = 96)**		
	**SA/A**	**N**	**D/SD**	**SA/A**	**N**	**D/SD**
**Quality of Life**						
I have no control over the situation	46(30)	21(14)	83(55)	36(38)	12(13)	48(50)
I just want to get it over with	79(53)	31(21)	40(27)	63(66)	12(13)	21(22)
Waiting has affected my quality of life	71(47)	34(25)	42(28)	56(58)	20(21)	20(21)
The length of waiting is a big concern	98(65)	28(19)	24(16)	73(76)	11(12)	12(13)
It is very stressful waiting for obesity treatment	79(53)	31(21)	40(27)	66(69)	14(15)	16(17)
My life has been put on hold while I wait for obesity treatment	43(29)	32(21)	75(50)	35(37)	17(18)	44(46)
Waiting costs you physically, mentally and financially	93(62)	30(20)	27(18)	68(71)	16(17)	12(13)
I am anxious and worried about treatment	67(45)	25(17)	57(38)	48(51)	14(15)	33(35)
There is no quality of life while waiting for treatment	32(22)	33(22)	84(56)	25(26)	20(21)	50(53)
**Employment Issues**						
Because of my weight problem, I am unable to work	21(14)	17(11)	112(75)	15(16)	11(12)	70(73)
Because of my weight problem, I can’t work a full shift	26(17)	12(8)	112(75)	20(21)	9(9)	67(70)
Money is a great issue for me now	68(46)	31(21)	50 (34)	45(47)	22(23)	29(30)
**Physical Stress**						
I make sure I don’t overdo things	96(64)	9(6)	45(30)	60(63)	6(6)	30(31)
Physical activities take me longer now	124(83)	8(5)	18(12)	87(91)	2(2)	7(7)
My activity is reduced because of my obesity	126(85)	11(7)	12(8)	91(96)	1(1)	3(3)
I can’t do many of the things I used to do	120(81)	12(8)	17(11)	87(92)	4(4)	4(4)
I am very short of breath	84(56)	17(11)	48(32)	65(68)	11(12)	19(20)
I have angina	7(5)	45(31)	95(65)	6(7)	36(39)	51(55)
The rest of my body is suffering because of my weight condition	129(86)	8(5)	13(9)	89(93)	4(4)	3(3)
My symptoms are getting worse	103(69)	23(15)	24(16)	75(78)	14(15)	7(7)
I am feeling fine now	52(35)	23(15)	75(50)	27(28)	13(14)	56(58)
I’m not sure what activity I can do without hurting my condition	60(41)	34(23)	55(37)	42(43)	24(25)	30(31)
I’ve stopped smoking recently	17(12)	55(37)	75(51)	13(14)	29(31)	53(56)
**Social Support**						
My family and friends are very patient and supportive	123(82)	15(10)	12(8)	79(82)	8(8)	9(9)
I try to cope	137(91)	10(7)	3(2)	94(98)	2(2)	0(0)
I have faith in the doctors	115(76)	19(13)	16(11)	73(76)	11(12)	12(13)
Waiting is very tough on my family and friends	68(45)	53(35)	29(19)	50(52)	28(29)	18(19)
I would attend a support group for people with obesity	111(74)	24(16)	15(10)	75(78)	14(15)	7(7)
A big factor is the lack of communication in the system	74(49)	55(37)	21(14)	50(52)	34(35)	12(13)
I would attend a class to learn more about obesity	121(81)	16(11)	13(9)	80(83)	8(8)	8(8)
**Frustration**						
It frustrates me that I have to wait for obesity treatment	121(81)	14(9)	15(10)	87(91)	3(3)	6(6)
I worry about what might happen while waiting (e.g. worsening symptoms, heart attack, death)	110(73)	19(13)	21(14)	85(89)	5(5)	6(6)
I’m frustrated with the allocation of resources	101(68)	32(22)	16(11)	71(75)	17(18)	7(7)
The problem with the waiting list is the allocation of resources	92(62)	42(28)	15(10)	66(70)	23(24)	6(6)
I am mad and upset about the wait	60(40)	52(35)	38(25)	49(51)	30(31)	17(18)
I am scared of treatment for obesity	57(38)	32(21)	60(41)	37(39)	20(21)	39(41)
I am afraid to go away from the phone for too long in case I miss a call for obesity treatment	12(8)	49(33)	89(59)	10(10)	34(35)	52(54)
The waiting list is not fair to everybody	64(43)	49(33)	36(24)	52(55)	29(31)	14(15)
I shouldn’t have to wait for obesity treatment	88(59)	33(22)	29(19)	65(68)	15(16)	16(17)

SA/A = strongly agree/agree; N = neutral; D = disagree/strongly disagree.

### Quality of life

The majority of subjects expressed concern over wait times (65%) and felt that waiting was very stressful (53%) and physically, emotionally and mentally taxing (62%).

### Employment

A minority of subjects indicated that they were unable to work (14%) or unable to work a full shift (17%). However, 46% of subjects still indicated that money was an issue in their lives.

### Physical stress

Physical limitations were common, with 85% reporting reduced activity, 83% reporting activity limitations compared to previous activity levels and 69% reporting worsening physical limitations over time.

### Social support

The majority of subjects reported a supportive social network (82%), faith in their physicians (76%) and trying to cope with waiting (91%). 74% indicated interest in attending a support group or classes to learn more about obesity.

### Frustration

Of the respondents, 81% of subjects indicated that the wait for care was frustrating, 73% worried about the consequences of extended wait times on their health, 68% were frustrated with the allocation of resources and 59% felt that they should not have to wait for obesity treatment.

### Satisfaction

Of the 150 subjects surveyed, 59% strongly agreed/agreed that the medical care they had received was ‘just about perfect’, 23% were uncertain, 16% disagreed/strongly disagreed, and 3% did not answer. Thirty-one percent of subjects were ‘dissatisfied with some things about the medical care they received’, 17% were uncertain, 49% disagreed/strongly disagreed, and 3% did not answer.

### Subgroup interested in bariatric surgery

Results were broadly similar in this subgroup, although greater impairments in the quality of life, frustration and physical stress domains of the WLIQ were apparent compared to the overall sample (Table 3). Patient satisfaction results were nearly identical to the overall sample (data not shown).

## Discussion

Most patients wait-listed for multidisciplinary bariatric assessment within a population-based regional bariatric program attributed impairments in overall health status and adverse health consequences to their time spent waiting. In particular, overall VAS scores were very low and the WLIQ scores indicated that the domains of quality of life, frustration, and physical stress were most affected, particularly in subjects interested in surgery. Nevertheless, the majority of patients were still satisfied with their medical care.

The number of patients who meet guideline-concordant eligibility for surgery is currently orders of magnitude greater than the capacity to perform this procedure and will likely rise further if contemporary trends in the prevalence of morbidly obesity continue [24,25]. Thus, only a fraction of eligible patients will realistically ever undergo a bariatric procedure. In 2009, an estimated 1.5 million Canadian adults were potentially eligible for bariatric surgery, yet only 1500 publically funded bariatric procedures (0.1% of eligible patients) were performed [11]. Applying similar calculations [9] to 2009-10 obesity prevalence figures from England, we estimate 3.3 million individuals were potentially eligible for bariatric surgery and yet only 3600 surgeries were performed (also equal to 0.1% of eligible patients) [7]. A recent economic analysis from England reported that expanding the provision of surgery from the current capacity to 5% of eligible patients would save £417 million over three years, with savings largely realized from increased productivity, reduced health care costs and reduced disability payments [7]. Yet, a survey of 23 Primary Care Trusts in England found that only 5 (23%) were planning to increase provision of surgery and 4 (17%) were planning to decrease the number of procedures performed [7]. In Canada, national surgical volumes from 2004-07 have remained flat, and although several provinces have recently announced plans to increase provision of surgery, others have either cut back or have decided against offering bariatric procedures to their populace [26]. These data suggest that large increases in the numbers of bariatric surgery performed within the public health care sector are unlikely to occur in the near future.

**Figure 2:**
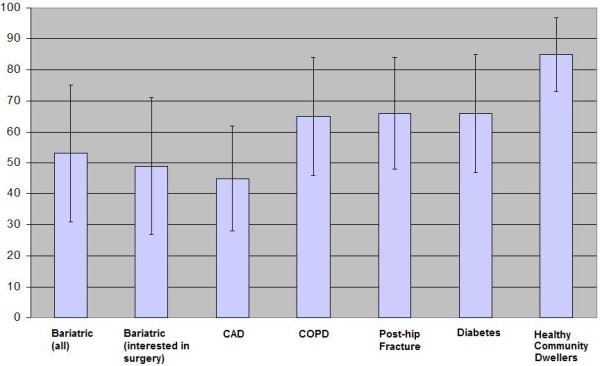
** Comparison of Visual Analogue Scores (VAS) in Different Patient Samples.** Error Bars depict standard deviations. VAS scores for non-bariatric samples taken from references 20 and 22. CAD = coronary artery disease; COPD = emphysema

The strikingly low VAS scores found in this sample indicate a substantial degree of self-reported health status impairment. The mean value of 49 is markedly lower than the average score of 85 previously reported in a random sample of community-dwelling adults drawn from the same population as our study sample [27]. In fact, the mean VAS scores in our bariatric population are considerably lower than those reported other chronic medical conditions (Figure 2) such as diabetes and COPD with VAS scores of 66 and 65 respectively [23,28,29].

To our knowledge, this is the first study to examine perceptions of the impact of extended wait times on health, patient satisfaction and VAS scores in wait-listed patients. We found only one other study examining QOL in severely obese patients wait-listed for bariatric care [30]. This Norwegian study reported that both the mental and physical components of the Short Form-12 (SF-12) were markedly lower in 128 severely obese patients compared to population norms. However, wait time duration, patients’ views regarding wait times and patient satisfaction were not assessed. Our findings are also similar to previous studies reporting lower quality of life compared to population controls in patients approved for and awaiting bariatric surgery and other elective procedures such as coronary bypass grafting and joint replacement [18,23,31,32]. However, unlike patients already approved for bariatric or other types of surgery, the patients we studied interested in surgery were still facing protracted multiyear wait times before even being assessed for this procedure. Given the association between poor self-reported health status and increased morbidity and mortality [32] and given that health status and quality of life improve after medical and surgical bariatric care [15-18], our results raise concern regarding the potentially detrimental (and previously under-appreciated) health ramifications of extended wait times for bariatric care.

It is also noteworthy that we identified higher BMI levels, unemployment and depression as statistically significant independent correlates of lower VAS scores. Beyond statistical significance, the magnitude of difference in health status between employed and unemployed individuals (12 points on the VAS) and depressed and non-depressed subjects (10 points) are clinically important [33]. These results suggest that support programs to help maintain workforce participation for bariatric patients may be of value – even while they await assessment and more definitive bariatric management. In addition, because protracted wait times are unlikely to disappear in Canada and similarly structured health systems, supportive interventions designed to treat depression and reduce the physical and psychological stress of waiting may improve health status and quality of life in wait-listed patients. Such interventions are ideally best delivered as an adjunct to ongoing non-surgical weight management efforts, although there are as yet no randomized trial data to support this contention. That said, a small randomised trial evaluating a nurse led, shared care monthly intervention consisting of health education and motivational interviews for wait-listed coronary artery bypass surgery candidates significantly improved cardiovascular risk factors as well as general health status, levels of depression, anxiety, and physical activity levels compared to usual care [34]. Similarly, improvements in quality of life, well-being and social support were reported in a telephone-based psychosocial intervention for patients awaiting lung transplantation [35]. Studies evaluating similar interventions in bariatric populations should be considered, although because of the large number of individuals wait-listed, group interventions would likely be required.

### Strengths and limitations

The main strengths of this work are that the sample was population-based, that the data were collected prospectively, and that it is the first investigation of its kind among patients wait listed for multidisciplinary bariatric assessment and management. We feel that the results of our study can be readily generalizable to other programs across Canada given the population-based nature of our data and the similarities between bariatric care delivery (especially for surgery) in other Canadian provinces. There are however several limitations. First, the cross-sectional design of our study limits our ability to measure changes in health status and satisfaction over time, but ongoing follow-up of the APPLES cohort will provide longitudinal data on these and additional outcomes [11]. Second, our population was surveyed early during their wait (within a few months of a multi-year process) and we cannot yet determine if health status and quality of life will further deteriorate or start to improve over time. Third, the survey participation rate was low at 35%, which limits the generalizability of the results to all wait-listed patients. However, we do note that there were no major differences in the demographic characteristics of those who participated and those who did not. Fourth, the sample was comprised primarily of women, limiting the generalizability of the results to males. However, the demographics (including the female sex preponderance and relatively high education and income levels) of our study sample are similar to nationally representative samples of patients undergoing bariatric care [36,37]. Fifth, although the WLIQ has been previously used in patients with coronary disease, there are no studies explicitly validating this instrument in morbidly obese patients.

## **Conclusions**

In conclusion, we have identified that patients wait listed for bariatric surgery report very impaired health status and that depression, unemployment and higher BMI predict greater health status impairment. Care providers and decision makers should consider the heretofore undescribed physical and psychological toll of waiting (and the ramifications with respect to quality and quantity of life) when devising strategies to optimally manage wait lists within publicly funded bariatric programs.

## Competing interests

AMS has received consultant fees and speaking honoraria from Allergan and Johnson and Johnson. DB has been an advisor and has received speaking honoraria and research funding from Johnson & Johnson Medical Products and Eithicon Endo-Surgery. The other authors declare no conflicts of interest with respect to this work.

## Authors’ contributions

RP and SRM developed the original study proposal with input from the other authors. Data analysis was performed by RP and by EPICORE® centre. RP had full access to the data and takes responsibility for the integrity of the data and accuracy of the data analysis. RP wrote the initial draft and this was critically revised by the other authors. All authors approved the final manuscript.

## Pre-publication history

The pre-publication history for this paper can be accessed here:

http://www.biomedcentral.com/1472-6963/12/139/prepub
